# Assessing the Impact of Roasting Temperatures on Biochemical and Sensory Quality of Macadamia Nuts (*Macadamia integrifolia*)

**DOI:** 10.3390/foods12112116

**Published:** 2023-05-24

**Authors:** Noluthando Noxolo Aruwajoye, Nana Millicent Duduzile Buthelezi, Asanda Mditshwa, Samson Zeray Tesfay, Lembe Samukelo Magwaza

**Affiliations:** 1 Discipline of Crop and Horticultural Science, School of Agricultural, Earth and Environmental Sciences, University of KwaZulu-Natal, Private Bag X01, Scottsville, Pietermaritzburg 3209, South Africa; 2 Department of Biology and Environmental Sciences, Sefako Makgatho Health Sciences University, P.O. Box 235, Medunsa, Ga-Rankuwa 0204, South Africa

**Keywords:** peroxide value, fatty acids, flavonoids, phenols, antioxidants activity, sensory evaluation

## Abstract

Depending on the temperature regime used during roasting, the biochemical and sensory characteristics of macadamia nuts can change. ‘A4′ and ‘Beaumont’ were used as model cultivars to examine how roasting temperatures affected the chemical and sensory quality of macadamia nuts. Using a hot air oven dryer, macadamia kernels were roasted at 50, 75, 100, 125, and 150 °C for 15 min. The quantity of phenols, flavonoids, and antioxidants in kernels roasted at 50, 75, and 100 °C was significant (*p <* 0.001); however, these kernels also had high levels of moisture content, oxidation-sensitive unsaturated fatty acids (UFAs), and peroxide value (PV), and poor sensory quality. Low moisture content, flavonoids, phenols, antioxidants, fatty acid (FA) compositions, high PV, and poor sensory quality—i.e., excessive browning, an exceptionally crunchy texture, and a bitter flavor—were all characteristics of kernels roasted at 150 °C. With a perfect crispy texture, a rich brown color, and a strong nutty flavor, kernels roasted at 125 °C had lower PV; higher oxidation-resistant UFA compositions; considerable concentrations of flavonoids, phenols, and antioxidants; and good sensory quality. Therefore, ‘A4′ and ‘Beaumont’ kernels could be roasted at 125 °C for use in the industry to improve kernel quality and palatability.

## 1. Introduction

According to [1,2], macadamia (*Macadamia integrifolia*) is the top-ranking nut in the world and is frequently consumed either roasted or as an ingredient in different confectionery items. The widespread intake of macadamia nuts may be attributed to its high nutritional value and alleged health benefits, which include reducing the risk of type 2 diabetes and cardiovascular disease [3,4,5]. These health advantages are linked to low cholesterol levels, a high oil content (69–78%) that is rich in monounsaturated fatty acids (80%), primarily oleic (60%) and palmitoleic (20%) acids, and antioxidants due to a number of phytochemicals and polyphenols also present in kernels [2,5,6,7,8,9]. The beneficial macronutrients protein, dietary fiber, vital minerals, vitamin E, and plant sterols are also abundant in macadamia [10]. Macadamia nuts are especially sensitive to hydrolytic and oxidative rancidity when they contain significant levels of free moisture because they have a high proportion of monounsaturated fatty acids [11,12].

According to [13,14], roasting is one of the best processing techniques for maintaining the quality of nuts and increasing their overall palatability. Kernels underwent sensory and chemical modifications as a result of this processing technique [15]. Through non-enzymatic reactions such as Maillard browning, it enhances the color, flavor, aroma, and texture of the nuts [16,17,18]. The pyrazines and pyridine compounds that give roasted nuts their characteristic brown color are byproducts of the Maillard reaction, which requires reducing sugars to react with amino acids to produce browning products [12,16,19].

The Maillard process occurs during roasting and produces a variety of volatile chemicals that give kernels their flavor and aroma [15,20]. According to [21], pyrazines, furans, and pyrroles are crucial elements of the scent of roasted kernels. Pyrazines are produced during heating by Strecker degradation and Maillard sugar–amine reactions [12]. Pyrazines feature nutty and roasted odors. Compared to raw kernels, roasted kernels have a crispier texture and a more delicate, distinctively nutty flavor that makes them more popular for consumption [13,22]. Additionally, roasting deactivates the oxidative enzyme system (lipoxygenic enzymes), lowers moisture content, and, as a result, gets rid of germs while minimizing degradative reactions such as lipid oxidation, which has antioxidant activity [18,22].

The sensory attributes, nutritional value, and lipid quality of nuts can all be impacted by roasting, despite the fact that roasting has numerous positive effects [23]. Furthermore, excessive roasting or high temperatures can promote lipid oxidation and non-enzymatic browning reactions, which can decrease the nutritional value of food by causing the loss of essential fatty acids, essential amino acids, and carbohydrates [23,24]. Additionally, this may hasten the occurrence of lipid oxidation, sometimes referred to as oxidative rancidity. According to [25], these chemical modification reactions may lead to the production of hazardous chemicals in nuts that may be harmful to consumers’ health. Due to their high fat content and susceptibility to oxidation, macadamia nuts require low roasting temperatures [5,26]. The nut industry defines mild temperature as a range of 30 to 180 °C, during which roasting is performed for 15 to 60 min using a variety of techniques, including infrared heating, radio frequency and microwave dielectric processes, commercial electrical ovens, and so on [22,23,27]. However, little is known about the effect of mild roasting temperatures on the sensory and chemical quality of macadamia kernels. Therefore, the objective of this study was to evaluate the impact of different roasting temperatures on the sensory and biochemical quality of macadamia nuts.

## 2. Materials and Methods

### 2.1. Macadamia Samples

‘Beaumont’ and ‘A4′, two hybrids of Australian-bred *Macadamia integrifolia* cultivars, were taken from Elliot Farm’s commercial orchards in Port Shepstone, KwaZulu-Natal, South Africa (latitude: 30°44′28′′ S, longitude: 30°27′17′′ E, and elevation: 36 m). According to standard industry procedure, nuts were harvested during the early season (May 2017) and the late season (June 2017). For further processing, approximately 20 kg of nuts from each cultivar were gathered on each sample day and brought to the Ukulinga Research Farm of the University of KwaZulu-Natal (UKZN).

### 2.2. Dehusking, Drying, and Cracking Process

At UKZN’s Ukulinga Research Farm, dehusking was carried out on the same day as harvesting using a 01-one-lane dehusker from WMC Sheet Metal Works in Tzaneen, South Africa. Nuts-in-shell (NIS) were dried using a mechanical convection oven (RY-EB-550, Rongyao factory, Wuhan, China) at the UKZN Postharvest Research Laboratory for three days in a row, starting at 35 °C on the first day, 38 °C for the second day, and 50 °C for the third day, according to a method described by [28] with minor modifications. To prevent quality deterioration, these drying temperatures were chosen in accordance with nut industry practices [22,29]. The moisture level of the dried nuts was monitored, and they were only taken out of the oven when it reached 1.5%. Based on the initial and final (dry) weights, the moisture content of nuts was determined. According to [30], dry basis (db) calculations of moisture content in nuts were made using mass changes. According to [31], the moisture content was calculated as follows:

(1) $${\mathrm{MC}}_{\mathrm{db}}=\frac{W_{i}-W_{d}}{W_{d}}\times 100$$

where MC_db_ is moisture content on a dry mass basis, and $W_{i}\;$ and $W_{d}\;$ are the initial and the dry sample weights, respectively. Additionally, using a commercial mechanical macadamia nutcracker (TZ-150 macadamia nut cracker, Alibaba Group Houlding (PTY) LTD, Hangzhou, China), macadamia nuts were mechanically cracked into wholes, halves, and bits. Only whole kernels were utilized in the experiment, and the shell and kernel were manually separated. The percentage of kernel recovery was computed and expressed as a percentage weight of the nut that is a kernel at 1.5% moisture content, with the rest being a shell (Equation (2)). Raw kernels are nuts that have been dried to a kernel moisture level of 1.5% for the purpose of dehusking and cracking [32].

(2) $$\mathrm{KR}=\frac{\mathrm{NIS}\;-\mathrm{Shell}}{\mathrm{NIS}}\times 100$$

where KR is kernel recovery and NIS is nut-in-shell.

### 2.3. Roasting Process

The roasting process was carried out in accordance with [19] with minor modifications. Roasting temperatures were also selected based on industry norms for nuts in order to prevent quality degradation [18,22,23]. ‘A4′ and ‘Beaumont’ were used as two distinct cultivars during the early and late harvesting seasons. Only complete kernels with no visible faults were used for sampling. The kernels were roasted using a convection oven (RY-EB-550, Rongyao manufacture, Wuhan, China) for 15 min at 5 different temperatures, i.e., 50, 75, 100, 125, and 150 °C, at the Department of Horticultural Science Postharvest Laboratory of UKZN, South Africa. Raw kernels were used as the benchmark. For postharvest storage trials, commercial brown paper bags were filled with a randomly selected set of kernels, each weighing 2 kg and consisting of 15 replicates of each treatment, cultivar, and harvest season. After roasting, the sensory quality of the kernels was immediately assessed. The same day, the roasted kernels were ground into a fine powder and stored at −20 °C for future analysis using a Brabantia-table blender (BBEK 1051, Massdiscounter (Pty) Limited, Johannesburg, South Africa).

### 2.4. Quantification of Peroxide Value

Using a Brabantia-table blender (BBEK 1051, Massdiscounter (Pty) Limited, Johannesburg, South Africa), kernels were ground into a fine powder after roasting. The amount of kernel oil was quantified from ground sample material using a method similar to that of [33] with a few minor adjustments. Three grams of ground kernel sample were placed in a test tube with hexane (9.0 mL) before the test tube was immersed in an ultrasonic bath (Labotec, Model No. 132, Labotec (PTY) LTD, Johannesburg, South Africa) for 10 min. The test tube’s residual received another 6 mL of hexane after the supernatant was vacuum-filtered. The test tube was then filled with the Buchner funnel after being left for 5 min. Following filtering, the supernatant was dried with 15 mL of hexane using a GenVac^®^ concentrator (SPScientific, Genevac LTD, Suffolk, UK) while maintaining the sample’s oil content. The remaining oil was measured and reported using its dry weight.

With a few minor adjustments, the peroxide value (PV) was calculated in accordance with [29]. In total, 20 mL of acetic acid/chloroform (3:2 *v*/*v*) was used to dissolve a 1 g sample of oil, and then a burette was used to add 1 mL of a saturated potassium iodide solution. Iodine was created after the reaction had occurred. Deionized water (50 mL) was added. Using a burette, iodine was titrated with Na_2_S_2_O_3_. The following formula was used to compute the peroxide value (expressed as milliequivalents of peroxide per kilogram of sample):

(3) $$\mathrm{PV}\;\left(\mathrm{meq}\;\mathrm{per}\;\mathrm{kg}\right)=\frac{\left(S\;-\;B\right)\times N\;\times 1000}{\mathrm{Sample}\;\mathrm{wt}\;\left(g\right)\times 1000}$$

where S = sample titration (µL); B = blank titration; N = normality of Na_2_S_2_O_3_.

### 2.5. Quantification of Fatty Acid Profile

With minor changes, the method described by [5,34] was used to determine fatty acids (FAs). First, 2 mL of hexane was added after the sample was weighed at 100 mg. As an internal standard, heptadecanoic (C17) was added (50 µL of 1000 ppm). The sample combination received 1 mL of 20% sulfuric acid in methanol solution. The mixture was then incubated for one hour in an oven that was kept at a temperature of 80 °C. The mixture was allowed to cool at room temperature after incubation. In order to extract the fatty acid methyl esters (FAMEs), 3 mL of 20% (*w*/*v*) NaCl was added. To facilitate phase separation, the materials were agitated firmly and then centrifuged. The upper hexane phase (hexane containing FAMEs) was transferred to a gas chromatograph (GC) vial.

GC (Trace1300, Thermo Scientific, Austin, Texas, USA) connected to a flame ionization detector (FID) was used to separate the FAMEs. A GC-FID system and a CTC Analytics PAL autosampler were connected. On a non-polar Stabil wax (60 m, 0.32 mm ID, 0.25 m film thickness), FAs were separated. In this experiment, 1 mL/min of helium was employed as the carrier gas. At 240 °C, the injector temperature was maintained. A 5:1 split ratio was used to inject 1 L of the material. The oven’s temperature was set to 50 °C for 2 min, then increased to 180 °C at a rate of 25 °C per minute and held for 2 min, 200 °C at a rate of 3 °C per minute and held for 5 min, and lastly 240 °C at a rate of 4 °C per minute and kept for 15 min. Using the Chrom-Card data system version 2.3 software for Windows (Thermo Electron, Rodano, Italy), each FA in the chromatogram was identified by comparing the retention times with certified standard mixes (Grain FAME Mix Supelco, Bellefonte, PA, USA; Catalog No: 47801) and quantified by the peak areas on the chromatogram. In terms of g of FA per g of total FAs, the results were expressed. The total fatty acids (TFA), the ratio of saturated to unsaturated fatty acids (SFA/MUFA), and the ratio of monounsaturated to polyunsaturated fatty acids (MUFA/PUFA) were all calculated using the fatty acid composition that was obtained [22].

### 2.6. Quantification of Free and Membrane-Bound Phenols

Phenols were identified using [35] as a guide, with a few minor modifications. One gram of each sample of roasted kernels was combined with ten milliliters of 99.8% (*v*/*v*) methanol and vortexed for thirty seconds. The mixture was then agitated for an additional night at room temperature in order to remove the free phenols. Following centrifugation of the mixture, the supernatant was filtered through Whatman^®^ no. 1 filter paper, and the sample was once again washed with 10 mL of solvent until the solvent was clear. The remaining kernel residue was then effectively released from cell wall-bound phenols using acid hydrolysis. Each sample received 10 mL of 60% aqueous methanol that had been acidified (2 M hydrochloric acid) before being submerged in a hot water bath for 90 min. Supernatants were filtered through a 0.45 m filter after being allowed to cool in glass tubes. Gallic acid monohydrate was used as a standard in the spectrophotometric determination of the total phenolic concentration, and the result was expressed as mg OF gallic acid equivalents (GAE) kg^−1^ of sample dry weight (5 mL of distilled water + 1 mL of extract + 1 mL Folin–Ciocalteu reagent + 10 mL of 7% Na_2_CO_3_ + 8 mL of distilled water, left at room temperature overnight).

### 2.7. Quantification of Total Flavonoids Concentration

Using quercetin as a standard, the total flavonoid concentration was measured using aluminum chloride (AlCl_3_) in accordance with an established procedure (kernel extract prepared for phenolic concentration determination). This procedure was based on that provided by [36], with a few minor adjustments. In a glass tube, 0.10 mL of the kernel extract was added. The reaction mixture was then added, and 5% NaNO_2_ (0.03 mL) was added after that. It was let to stand at room temperature for 5 min. Then, 0.03 mL of 10% AlCl_3_ was added and allowed to react for 6 min before being mixed with 0.2 mL of 1 mM NaOH and diluted to 1 mL with purified water. At 510 nm, the absorbance of the reaction mixture was measured in comparison to methanol, which served as a blank. In terms of sample dry weight, the results were represented as mg quercetin kg^−1^.

### 2.8. Quantification of Total Antioxidants Activity

#### 2.8.1. Free Radical Scavenging Capacity

The 2,2′-diphenyl-1-picrylhydrazyl (DPPH) free radical scavenging activity was assessed with some minor modifications from [37]. First, 1.94 mg of DPPH was dissolved in 50 mL of methanol to create the stock solution of the radical, which was then stored at −20 °C until usage. The DPPH stock solution was diluted with methanol to produce the working solution for the radical, which had an absorbance of around 0.98 (0.02) at 517 nm. In a polystyrene 4.5 mL cuvette, 20 L of gallic acid or sample extract was measured. Following the addition of 1 mL of 0.1 mM DPPH solution, which was carried out in the dark and covered with aluminum foil, the mixture received 800 L of 100% methanol and was left to remain at room temperature for 60 min. The results were represented as mg DPPH GAE kg^−1^ of sample dry weight after the absorbance was measured at 517 nm against an absolute methanol blank in low light.

#### 2.8.2. 2,2′-Azinobis-3-ethylbenzothiazoline-6-sulfonic Acid (ABTS) Assay

The 2,2′-azinobis-3-ethylbenzothiazoline-6-sulfonic acid (ABTS) concentration was calculated using [38] with a few minor adjustments. For evaluating the hydrophilic and lipophilic antioxidant fractions, respectively, a 7 mM solution of 2,2′-azinobis-3-ethylbenzothiazoline-6-sulfonic acid was made. The 7 mM ABTS solution was combined with 2.45 mM ammonium persulfate to create the ABTS radical cation (ABTS.+), which was then left to sit in the dark at room temperature for 3 to 6 h. After that, 10 mL of sample solution made from roasted material extracts in acetate buffer (pH 4.0) was added together with 1.0 mL of activated ABTS solution (A734 nm = 0.700 ± 0.5). The decrease in absorbance at 734 nm was recorded after 6 min and the results were expressed as mg ABTS GAE kg^−1^ of sample dry weight.

### 2.9. Sensory Evaluation

A randomized set of macadamia kernels were examined raw and immediately after roasting (50, 75, 100, 125 and 150 °C at the Department of Horticultural Science Postharvest Laboratory of UKZN. Samples were given to trained panelists ranging from the age of 25 to 44 to assess the quality of ‘A4′ and ‘Beaumont’kernels in terms of their texture, colour and taste [13,26,39]. The trained panelists employed in this study were 5 males and 5 females as described by similar studies where the panelists ranged between 7 and 12 in number [40,41,42]. Moreover, according to [13,39] with some minor modifications, the texture, colour, and taste of the kernels were evaluated using a 5-point scale as follows:Texture, where 1 denotes very hard, 2: hard, 3: slightly crispy, 4: crispy, and 5: very crispy; Colour, where 1 denotes very light, 2: light, 3: slightly brown, 4: brown, and 5: extremely brown and; Taste, where 1 denotes very nutty, 2: nutty, 3: slightly bitter, 4: bitter, and 5: extremely bitter.

### 2.10. Statistical Analysis

The collected data were subjected to analysis of variance (ANOVA) using GenStat statistical software (GenStat^®^, 18.1 edition, VSN International, Hemel Hempstead, UK) 18.1. Least significant difference values (LSD; *p* < 0.001) were calculated for mean separation.

## 3. Results and Discussion

### 3.1. Moisture Content

**Figure 1:**
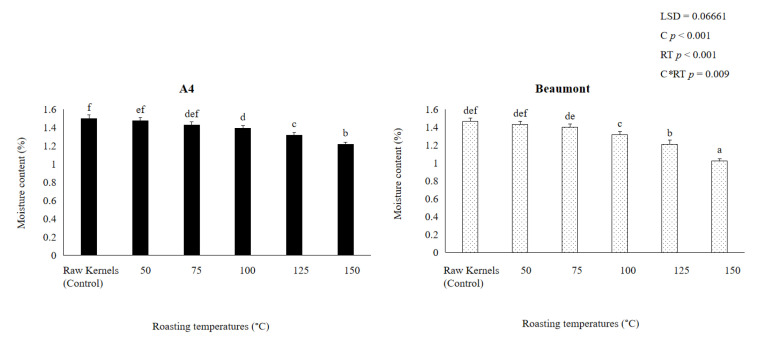
Moisture content of ‘A4′ and ‘Beaumont’ at different roasting temperatures. Different lowercase letters (a–f) indicate significant differences (*p* < 0.01) between the different roasting temperatures.

The ‘A4′ and ‘Beaumont’ cultivars’ kernel moisture content considerably (*p* < 0.001) reduced as the roasting temperature rose (Figure 1). ‘A4′ and ‘Beaumont’ kernels roasted at 50, 75, 100, and 125 °C exhibited substantial drops in moisture content of 1.474% and 1.432%, 1.429% and 1.4%, 1.393% and 1.318%, and 1.319% and 1.211%, respectively, whereas kernels roasted at 150 °C had the lowest moisture content of 1.218% and 1.025%, respectively. This might be caused by convectional roasting processes, where moisture is first removed from the kernel’s surface and then lost when water diffuses from the kernel’s inside to the dried surface [43]. Additionally, excessive moisture evaporation and the loss of volatile compounds may be due to the significant decrease in kernel moisture content at 150 °C. This problem is made worse by the reaction of free amino acids and short-chain peptides with free mono- and disaccharides during nonenzymatic browning, as well as the potential for protein denaturation and degradation when roasting nuts at temperatures above 130 °C [27]. These chemical alterations may have an impact on the sensory and chemical quality of the kernel [18,22,44]. As a result of using higher temperatures (150 °C) during roasting, Refs. [13,14,26] reported similar significant decreases in kernel moisture content from 2.4% to 1%.

### 3.2. Peroxide Value (PV)

The most-used metric to assess the degree of primary oxidation products, mostly hydroperoxides in edible oils, is the peroxide value [12,23]. According to [25,29], a number of variables, such as roasting temperature, levels of unsaturated fatty acids, the presence of enzymes, and antioxidant levels, may have an impact on the peroxide value, which is a sign of autoxidation (free radical reaction). Nut rancidity results from a reaction between the hydroperoxides produced by autoxidation and other nut components such as proteins and amino acids [45].

**Figure 2:**
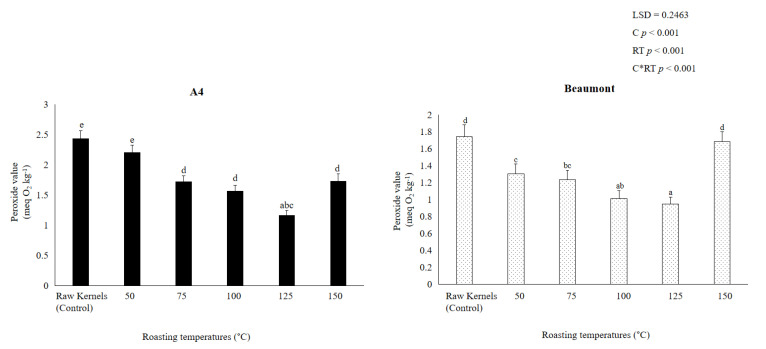
Peroxide value of ‘A4′ and ‘Beaumont’ at different roasting temperatures. Different lowercase letters (a–e) indicate significant differences (*p* < 0.01) between the different roasting temperatures.

From Figure 2, it can be observed that the PV of ‘A4′ and ‘Beaumont’ kernels roasted at 50 °C (2.203 and 1.303 meq O_2_ kg^−1^), 75 °C (1.72 and 1.232 meq O_2_ kg^−1^), 100 °C (1.569 and 1.011 meq O_2_ kg^−1^), and 125 °C (1.161 and 0.947 meq O_2_ kg^−1^) significantly (*p* < 0.001) declined with increasing roasting temperatures. It is thought that kernels roasted at 125 °C have reduced PV, which is a sign of high quality. A high-quality macadamia product is defined by a low peroxide value of less than 1 meq O_2_ kg^−1^, which is deemed acceptable for a freshly refined fat and is categorized by a low oxidation state, according to [24,46]. Our findings are in line with those of [45], who found that when roasting temperature increased (from 110 to 120 °C), the PV of Canariumindicum L. (Burseraceae) kernels drastically dropped from 1.30 to 1 meq O_2_ kg^−1^.

Both cultivars ‘A4′ (1.727 meq O_2_ kg^−1^) and ‘Beaumont’ (1.682 meq O_2_ kg^−1^) saw an increase in PV after being roasted at 150 °C. This could be attributable to greater temperatures, which encourage the peroxide’s breakdown and polymerization into a variety of secondary compounds such as aldehydes and ketones [47]. According to [25], temperature has a significant impact on the rate at which edible oils oxidize and produce rancidity; the higher the temperature, the faster rancidity develops. Our findings concur with those of [23,44], who claimed that the increase in PV caused by thermal processing in sesame and walnuts, respectively, could be attributed to the rise in hydroperoxides caused by free radicals attacking the unsaturated fatty acids of oil. Our results indicate that roasting ‘A4′ and ‘Beaumont’ kernels at 150 °C should be avoided since [24,47] showed that the increase in PV may result in the development of detectable off-flavors.

### 3.3. Fatty Acids (FAs)

The FAME analysis of raw (control) and roasted kernels of ‘A4′ and ‘Beaumont’ macadamia nuts identified a total of thirteen individual FAs, among which oleic acid (C18:1n9c) and palmitoleic acid (C16:1) were predominant, followed by palmitic acid (C16:0), omega-6 (n-6), linoleic acid (C18:2n6c), stearic acid (C18:0), and cis-11-Eicosenoic acid (C20:1) (Table 1 and Table 2). Additionally, Table 1 and Table 2 display the total amount of fatty acids (TFAs) found in ‘A4′ and ‘Beaumont’ kernels. ‘A4′ had the highest concentration of TFAs. The majority of the fatty acids in both cultivars were monounsaturated fatty acids (MUFA), followed by polyunsaturated fatty acids (PUFAs) and saturated fatty acids (STAs). Moreover, (∑ n-6)/(∑ n-3) was high in both cultivars. The ratio of ∑ PUFA:∑ SFA was low in both cultivars. These findings are in agreement with [5,22], who reported these major FAs in raw and roasted kernels of macadamia nuts and hazelnuts, respectively.

Table 1 and Table 2 show that control kernels of ‘A4′ and ‘Beaumont’ significantly (*p* < 0.001) had the highest amounts of ∑ TFAs (1458.25 and 1165.55 µg/g), ∑ MUFAs (1250.10 and 959.68 µg/g), ∑ PUFAs (71.75 and 68.39), (∑ n-6)/(∑ n-3) (97.82 and 113.77 µg/g), linoleic acid (C18:2n6c) (64.34 and 52.70 µg/g), dihomo-γ-linolenic acid (C20:3n6) (5.26 and 15.22 µg/g), and omega-6 (n-6) (69.61 and 67.92 µg/g), respectively. The kernels are nutritious due to the large number of unsaturated fatty acids (UFAs), but they are also more prone to lipid peroxidation and rancidity, particularly when they have a high moisture content [5,48]. High kernel moisture content stimulates the breakdown of lipids into free fatty acids (FFAs), which leads to the creation of rancidity, as well as the growth of microfungi that produce deteriorating enzymes [49]. This is supported by Figure 1 and Figure 2, which demonstrate that the control kernels of ‘A4′ and ‘Beaumont’ had significantly (*p* < 0.001) greater PV (2.432 and 1.741 meq O_2_ kg^−1^) and moisture content (1.5 and 1.46%), respectively. These values are within the acceptable range but indicate kernel susceptibility to the development of rancidity [11,48]. Moreover, control kernels of ‘A4′ and ‘Beaumont’ had significantly (*p* < 0.001) high ∑ SFAs (136.40 and 137.48 µg/g) and lower ∑ PUFA: ∑ SFA (0.43 and 0.38 µg/g), respectively. Our findings are similar to [50], who reported a low ratio of ∑ USFA: ∑ SFA (0.11%) and high ∑ SFA (8.42%), attributed to the low content of linoleic acid content, probably as the result of its peroxidation [51].

With higher roasting temperatures, the fatty acid contents of ‘A4′ and ‘Beaumont’ kernels significantly (*p* < 0.001) reduced (Table 1 and Table 2). This could be explained by the enzymes becoming inactive during roasting, which also results in a decrease in the activity of the lipase enzyme and moisture content [52]. Additionally, ‘A4′ and ‘Beaumont’ kernels roasted at 150 °C demonstrated a further drop and a smaller number of FA components. According to [52], excessive roasting at temperatures above 150 °C could hasten the oxidation of lipids. ‘A4′ and ‘Beaumont’ kernels roasted at 150 °C had considerably (*p* < 0.001) lower levels of linoleic acid (C18:2n6c) of 0.01 and 0.09 g/g, respectively. According to [43], the breakdown of linoleic acid during heating processes might result in the production of aliphatic aldehydes such as 2-heptenal and nonanal, which are to blame for the emergence of bad flavors and odors in nuts [49]. According to the Southern African Macadamia Growers Association (SAMAC), 1.73 and 1.69 meq O_2_ kg^−1^ of the ‘A4′ and ‘Beaumont’ kernels, respectively, roasted at 150 °C had significantly (*p* < 0.001) higher PV, which is within the acceptable limit, but illustrates the vulnerability of kernels to the development of rancidity [48].

Additionally, ‘A4’ and ‘Beaumont’ kernels roasted at 125 °C exhibited considerably (*p* < 0.001) the largest concentrations of oleic acid (C18:1n9c) (751.97 and 624.92 g/g) and palmitoleic acid (C16:1) (472.96 and 340.36 g/g), respectively. This may be because some FAs are released when kernels are roasted at 125 °C, resulting in the synthesis of Maillard and caramelization browning products [18]. Oleic acid-rich oils are less likely to experience lipid oxidation [53]. ‘A4’ and ‘Beaumont’ kernels roasted at 125 °C contained significantly (*p* < 0.001) more oleic acid (C18:1n9c) (800.97 and 624.92 ug/g) and lower linoleic acid (C18:2n6c) (32.74 and 22.02 ug/g), respectively. Higher temperature during roasting can result to degradation of unsaturated fatty acids [54]. Ref. [55] observed from their study on the stability and change in fatty acids composition of several oils that oils rich in linoleic and linolenic acids are less heat-tolerant compared to those rich in oleic acid (C18:1). In their study, the oils rich in oleic acid remained relatively stable during heat treatment temperatures between 150 and 180°C [55]. Thus, this could be the case for the Oleic Acids and lower linolenic acids observed when nuts were roasted at 125 °C.

Moreover, ‘A4’ and ‘Beaumont’ kernel roasted at 125 °C are less prone to lipid oxidation and the formation of rancidity. ‘A4’ and ‘Beaumont’ kernels roasted at 125 °C had significantly (*p* < 0.001) reduced PV (1.16 and 0.95 meq O_2_ kg^−1^, respectively), proving this. These PV show that the kernels are fresh and do not taste rancid because they are within the minimal permissible level (1–3 meq O_2_ kg^−1^) [11,48].

### 3.4. Phenolic Concentration

**Figure 3:**
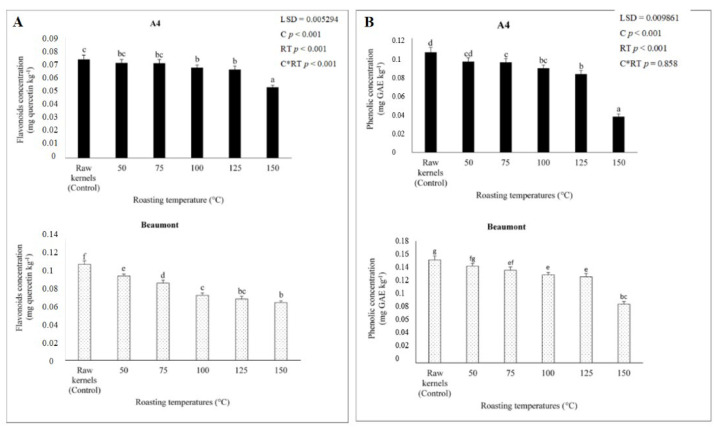
Flavonoid (**A**) and phenolic (**B**) concentrations of ‘A4′ and ‘Beaumont’ kernels at different roasting temperatures. Different lowercase letters (a–g) indicate significant differences (*p* < 0.01) between the different roasting temperatures.

Phenolic concentration was discovered to be inversely correlated with flavonoid concentration, with phenolic concentrations of ‘A4′ and ‘Beaumont’ roasted at 50 °C (0.096 and 0.143 mg GAE kg^−1^), 75 °C (0.095 and 0.137 mg GAE kg^−1^), 100 °C (0.089 and 0.130 mg GAE kg^−1^), 125 °C (0.082 and 0.127 mg GAE kg^−1^), and 150 °C (0.038 and 0.087 mg GAE kg^−1^). The majority of phenolic chemicals, according to [56], are extremely unstable and may be lost during processing. In accordance with [44], who reported that roasting temperatures above 130 °C lead to a gradual decrease in total phenolic concentration as a result of thermal and oxidative degradation of polyphenols and intermediate products of the brown reaction, it can be seen from Figure 3 that roasting ‘A4′ and ‘Beaumont’ kernels at 150 °C further reduced phenolic concentration. In pine nuts roasted at 150 °C, Ref. [52] similarly found a comparable decline in total phenolics, which they hypothesized would be caused by heat stress-induced thermal breakdown of phenolic compounds. Additionally, Ref. [57] observed that rancidity, oxidation, and hydrolysis could all contribute to the breakdown of phenols at high roasting temperatures. This confirms our findings even more, as Figure 2 demonstrates that kernels roasted at 150 °C had high PV within the permissible range, but also suggests that these kernels are more prone to developing rancidity [24].

### 3.5. Flavonoid Concentration

With increasing roasting temperature, the flavonoid concentrations of ‘A4′ and ‘Beaumont’ kernels decreased significantly (*p* < 0.001) (0.071 and 0.097 mg quercetin kg^−1^, 0.071 and 0.089 mg quercetin kg^−1^, 0.067 and 0.075 mg quercetin kg^−1^, 0.066 and 0.071 mg quercetin kg^−1^, and 0.053 and 0.067 mg quercetin kg^−1^). According to [58], depending on the temperatures used to roast items, the concentration of flavonoids in food and their functional qualities may change during thermal processing [59,60]. According to [16], some antioxidants are lost during roasting as a result of chemical processes such as the Maillard browning reaction. In our study, roasting the ‘A4′ and ‘Beaumont’ kernels at 150 °C further decreased the flavonoid content (Figure 3). This may also be due to the kernels’ vulnerability to rancidity after being roasted at 150 °C, as shown by their high PV levels (Figure 2), which may encourage nutritional loss and the emergence of bad flavors [61]. Given that the synthesis and polymerization of 5-hydroxymethylfurfural (HMF) are accelerated at low moisture content, the drop in flavonoid concentration at 150 °C may also be attributable to hydrolytic mechanisms. This is consistent with our findings, as Figure 1 demonstrates that the lowest moisture level was found in kernels roasted at 150 °C, which may have facilitated the loss of flavonoids during roasting. According to [62], high roasting temperatures may cause intracellular water to evaporate, resulting in significant changes in the chemical composition, including the formation of protein, amino acids, reducing sugars, sucrose, trigonelline, chlorogenic acid, and melanoidins, which are primarily caused by Maillard reactions. The loss of flavonoids could come from several chemical processes. Our results are consistent with those of [60], who found that apricot kernels roasted at 720 W (24.56 mg CE/g DW) had lower flavonoid concentrations than those roasted at 540 W (32.41 mg CE/g DW), presumably due to heat stress.

### 3.6. Antioxidant Activity (DPPH (2,2′Diphenyl1-picrylhydrazyl) and ABTS (2,2′-Azinobis-3-ethylbenzothiazoline-6-sulfonic Acid) Assay)

**Figure 4:**
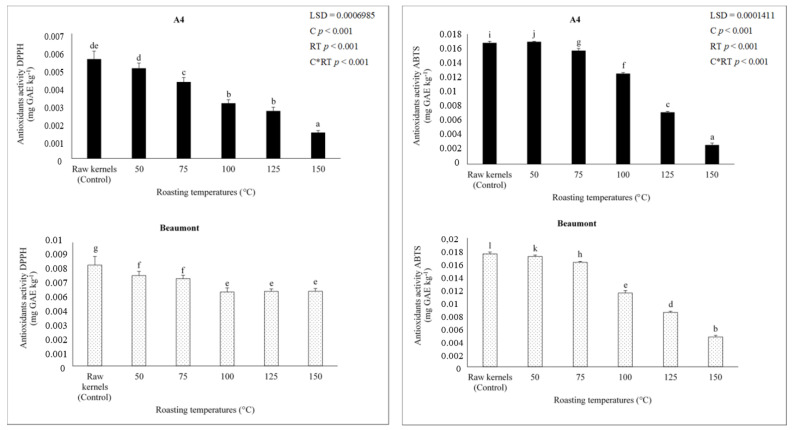
Antioxidant activity of ‘A4′ and ‘Beaumont’ at different roasting temperatures. Different lowercase letters (a–l) indicate significant differences (*p* < 0.01) between the different roasting temperatures.

The decrease in flavonoids and phenols was accompanied by changes in antioxidant activity (Figure 3). ‘A4′ and ‘Beaumont’ cultivars’ antioxidant activity (DPPH and ABTS) significantly (*p* < 0.001) decreased with rising roasting temperature. Compared to kernels roasted at a higher temperature of 150 °C (0.003 and 0.005 ABTS mg GAE kg^−1^ and 0.001 and 0.006 DPPH mg GAE kg^−1^, respectively), ‘A4′ and ‘Beaumont’ kernels roasted at 50 °C had a high concentration of antioxidants (0.017 and 0.017 ABTS mg GAE kg^−1^ and 0.005 and 0.007 DPPH mg GAE kg^−1^). Ref. [63] reported a similar decrease in antioxidants in hazelnuts roasted at 180 °C, which may be due to the oxidation of phenols [64]. It is clear from Figure 4 that roasting ‘A4′ and ‘Beaumont’ kernels at 150 °C further reduced the antioxidant activity. Juhaimi et al. [60] showed that apricot kernels roasted at 720 W had decreased antioxidant activity, which may have been caused by phenolics being damaged by heat. Higher roasting temperatures, such as 160 °C, promote excessive browning in products commonly referred to as dark roast in beans and further reduce the total antioxidant activity due to oxidation, hydrolysis, decarboxylation, and other degradative chemical reactions, resulting in the degradation of the product’s sensory and chemical quality, according to [65,66]. This further corroborates our observations because kernels roasted at 150 °C displayed a notable rise in PV (Figure 2).

### 3.7. Sensory Evaluation

#### 3.7.1. Kernel Texture

**Figure 5:**
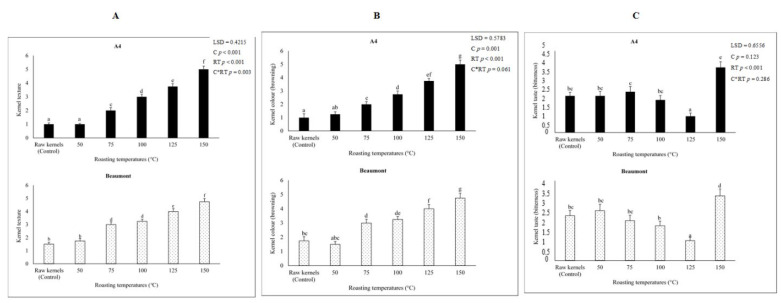
Kernel texture (**A**), color (**B**), and taste (**C**) of ‘A4′ and ‘Beaumont’ at different roasting temperatures. Different lowercase letters (a–g) indicate significant differences (*p* < 0.01) between the different roasting temperatures.

As the roasting temperature increased, ‘A4′ and ‘Beaumont’ kernels became considerably (*p* < 0.001) more crispy, which is thought to be a fundamental property of roasted kernels [67]. This might be a result of heat permeating the kernels, which would reduce moisture content and make the kernels crispy [68]. According to [69,70], the internal microstructure of samples is altered during roasting, producing a texture that is typically more brittle, crispy, and/or crunchy. The appealing crispy texture of the roasted kernels at 125 °C won the panelists’ favor. Our results are consistent with those of [71], who said that eight trained panelists preferred pistachio kernels that had been roasted at 120 °C because such kernels had the most appetizing crispy texture. According to [71,72,73], pistachio and soybean kernels roasted at lower temperatures (100 °C) had a hard texture that is less crispy. Figure 5A also demonstrates that kernels roasted at lower temperatures (50, 75, and 100 °C) had a hard texture. Heat stress caused unattractive excessive crispiness in ‘A4′ and ‘Beaumont’ kernels when they were roasted at 150 °C [13].

#### 3.7.2. Kernel Color

Because brown pigments increase as the Maillard and caramelization reactions advance, color is one of the criteria that is employed for process control during roasting [71]. In addition, it serves as one of the most crucial characteristics of roasted nuts and a gauge of kernel flavor [67,68,74]. In our study, the panelists rated the color of the roasted kernels as poor because they had a light color, whereas roasting temperatures over 75 °C considerably (*p* < 0.001) improved the color of the kernels (Figure 5). The development of color due to pyrazines and pyridines compounds, which are recognizable products resulting from non-enzymatic browning reactions such as Maillard browning and caramelization, could be attributed to the browning of ‘A4′ and ‘Beaumont’ kernels with increasing temperatures [12,28,75]. Le Lagadec et al. [76] added that enzymatic and non-enzymatic processes that take place during roasting transform sucrose into the reducing sugars glucose and fructose. The Maillard reaction, which appears to be responsible for the browning of the kernels, is primarily made up of reducing sugars and amino acids [19]. According to our study, roasted kernels at 125 °C had the most desired brown color, whereas those at 150 °C were overly brown and therefore over-roasted [77]. Our results are consistent with those of [78], who found that roasting almond nuts over 150 °C caused the kernels to become darker in color. This darker color may be the consequence of a non-enzymatic reaction that happens when a reducing sugar and protein are cooked together [79].

#### 3.7.3. Kernel Taste

The panelists felt that lower roasting temperatures of 50 and 75 °C lead to mildly nutty-tasting kernels, while roasting temperatures above 75 °C considerably (*p* < 0.001) improved kernel flavor (Figure 5). ‘A4′ and ‘Beaumont’ kernels roasted at 100 °C had a nutty taste, and kernels roasted at 125 °C had an extremely nutty taste, which might be related to the development of desired flavors through Maillard reactions during roasting [18,67]. According to [71], the chemical reactions that take place during roasting, such as the interaction of carbohydrates with proteins, lipids, and physiologically active compounds, result in the production of flavor and aroma. According to [17,76,80], benzaldehyde, methylphenol, and alkylbenzenes are flavor and aromatic chemicals that are produced during roasting and provide nuts a more palatable, improved, and robust nutty-roasted flavor. Additionally, kernels roasted at 125 °C had the lowest PV, indicating that there were no discernible off-flavors in the kernels [11]. Although [71] showed that higher roasting temperatures (150 °C) induced an increase in bitterness in pistachio kernels, the ‘A4′ and ‘Beaumont’ kernels were assessed as being extremely bitter. High roasting temperatures cause proteins and carbohydrates to alter, lipids to oxidize, and the flavor of kernels to become bitter [71]. High roasting temperatures (>150 °C), according to [81], impairs the stability of the hazelnuts’ proteins, carbohydrates, and oil. This reduces the overall palatability of the products and makes the kernels bitter. These findings are similar to those of Ref. [68], who observed that roasting peanuts at high temperatures (149 to 204 °C) caused a bitter taste because these temperatures cause the oxidation of secondary products such as aldehydes, ethanol, and ketones, which are linked to off-flavors. Additionally, kernels roasted at 150 °C had higher PV levels (Figure 2), an excessively crispy texture (Figure 5), and a dark brown color, all of which are signs of over-roasting and may lead to the development of unsavory compounds such as 5-hydroxymethylfurfural, which is linked to off-flavors or a bitter taste [18,71].

## 4. Conclusions

According to the results of the current study, although ‘A4′ and ‘Beaumont’ kernels roasted at low temperatures (50, 75, and 100 °C) had slightly elevated levels of flavonoids, phenols, and antioxidants, they also had higher moisture levels and FA composition, which promoted the development of rancidity, as indicated by higher levels of PV and poor kernel texture, color, and taste, as noted by trained panelists. The chemical and sensory qualities of macadamia nuts were greatly impacted by increasing roasting temperatures. ‘A4′ and ‘Beaumont’ kernels roasted at 125 °C had lower levels of rancidity (PV), high levels of oxidation-resistant FA compositions such as oleic acid, which delayed the rancidification process, and low levels of FA compositions, such as linoleic acid, which accelerated the oxidation process. Flavonoids, phenols, and antioxidants were significantly concentrated in the roasted kernels at 125 °C as well. Additionally, the judges said that roasted kernels at 125 °C had a perfectly crisp texture, a brown color, and were very nutty. However, a significant amount of rancidity; decreased content of flavonoids, phenols, and antioxidants; and an excessively crispy texture, a dark brown color, and a bitter flavor were present in kernels that were roasted at 150 °C. The results of this study, therefore, recommend roasting ‘A4′ and ‘Beaumont’ kernels at 125 °C to prevent rancidity and quality loss due to over-roasting. Additionally, because macadamia kernels quickly acquire rancidity, it is not advised to keep them fresh.

## Figures and Tables

**Table 1:** Fatty acid compositions (µg/g) of ‘A4′ macadamia kernels with different roasting temperature treatments.

RT	Lauric Acid (C12:0)	Miristic Acid (C14:0)	Palmitic Acid (C16:0)	Stearic Acid (C18:0)	Arachidic Acid (C20:0)	Palmitoleic Acid (C16:1)	Oleic Acid (C18:1n9c)
Control	1.99 ± 0.03 a	8.43 ± 0.58 c	83.31 ± 44.22 b	42.67 ± 5.92 b	27.25 ± 4.08 c	469.71 ± 26.86 c	709.66 ± 56.78 c
50 °C	1.91 ± 0.67 a	7.06 ± 0.71 bc	93.01 ± 28.95 b	33.75 ± 2.97 ab	20.98 ± 0.40 bc	384.59 ± 24.75 b	571.27 ± 28.00 b
75 °C	1.89 ± 0.09 a	7.03 ± 0.85 bc	94.29 ± 8.58 b	33.77 ± 818 ab	16.48 ± 3.07 ab	286.80 ± 33.47 a	496.32 ± 25.79 b
100 °C	2.69 ± 1.08 ab	6.40 ± 0.57 ab	94.10 ± 2.74 b	22.03 ± 2.89 a	16.74 ± 1.07 ab	248.90 ± 33.61 a	395.45 ± 28.17 a
125 °C	1.49 ± 0.06 a	5.33 ± 0.44 ab	96.28 ± 13.37 bc	21.22 ± 3.09 a	13.81 ± 1.77 ab	472.96 ± 13.79 c	800.97 ± 13.64 d
150 °C	1.45 ± 0.08 a	4.67 ± 0.400 a	63.71 ± 9.67 a	20.54 ± 2.27 a	12.22 ± 2.65 a	214.16 ± 17.37 a	357.42 ± 17.37 a
RT	Cis-11-Eicosenoic acid (C20:1)	Linoleic acid (C18:2n6c)	Dihomo-γlinolenic acid (C20:3n6)	Eicosatrienoic acid (C20:3n3)	Omega-6 (n-6)	Omega -3 (n-3)	(∑ n-6)/(∑ n-3)
Control	43.48 ± 5.92 c	64.34 ± 5.54 c	5.26 ± 0.96 c	2.14 ± 0.89 b	69.61 ± 6.45 d	2.14 ± 0.89 b	97.82 ± 46.57 c
50 °C	32.91 ± 1.72 bc	53.54 ± 6.52 bc	2.58 ± 0.40 b	1.05 ± 0.34 a	56.12 ± 6.21 cd	1.05 ± 0.34 ab	86.34 ± 34.47 c
75 °C	27.22 ± 1.87 ab	42.59 ± 1.21 ab	2.02 ± 0.57 b	0.83 ± 0.31 a	44.62 ± 1.04 bc	0.83 ± 0.31 ab	94.63 ± 39.57 c
100 °C	ND	34.67 ± 1.81 a	1.30 ± 0.13 ab	ND	34.25 ± 0.86 a	ND	ND
125 °C	22.68 ± 1.43 a	32.74 ± 1.89 a	1.38 ± 0.54 ab	1.55 ± 0.08 b	34.80 ± 1.88 ab	0.88 ± 0.08 ab	23.31 ± 1.14 a
150 °C	22.51 ± 1.17 a	33.83 ± 3.05 a	0.01 ± 0.60 a	1.56 ± 0.27 b	35.74 ± 3.08 ab	0.91 ± 0.27 ab	44.58 ± 12.77 b
RT	∑ PUFA:∑ SFA	∑ SFA	∑ MUFA	∑ PUFA	∑ TFA		
Control	0.65 ± 0.17 ab	136.40 ± 39.87 c	1250.10 ± 50.67 d	71.75 ± 7.21 c	1458.25 ± 37.18 e		
50 °C	0.48 ± 0.11 a	135.69 ± 27.83 c	1009.74 ± 28.97 c	57.17 ± 6.20 b	1202.61 ± 15.31 d		
75 °C	0.36 ± 0.03 a	127.74 ± 18.06 c	813.21 ± 25.38 b	45.45 ± 1.25 ab	986.40 ± 16.01 c		
100 °C	0.39 ± 0.02 a	92.72 ± 3.56 b	650.84 ± 16.37 a	35.64 ± 1.56 a	779.20 ± 17.30 b		
125 °C	0.55 ± 0.07 a	73.04 ± 11.84 a	651.27 ± 6.62 a	34.75 ± 1.93 a	631.51 ± 10.81 a		
150 °C	0.43 ± 0.08 a	90.41 ± 9.10 b	700.68 ± 19.24 ab	36.42 ± 3.28 ab	727.52 ± 14.62 b		

RT (roasting temperature), ∑ (sum), SFA (saturated fatty acids), MUFA (monounsaturated fatty acids), PUFA (polyunsaturated fatty acids), PUFA:SFA (ratio of polyunsaturated fatty acids to saturated fatty acids), TFA (total fatty acids), ND (not detected). Values are the mean ± SE. Means within a column of the same parameter with different letters are significantly different (*p* < 0.001).

**Table 2:** Fatty acid compositions (µg/g) of ‘Beaumont’ macadamia kernels with different roasting temperature treatments.

RT	Lauric Acid (C12:0)	Miristic Acid (C14:0)	Palmitic Acid (C16:0)	Stearic Acid (C18:0)	Arachidic Acid (C20:0)	Palmitoleic Acid (C16:1)	Oleic Acid (C18:1n9c)
Control	1.88 ± 0.16 a	7.61 ± 1.56 bc	94.27 ± 19.48 b	33.72 ± 4.76 bc	13.90 ± 2.75 b	336.75 ± 27.80 d	602.10 ± 45.62 de
50 °C	1.94 ± 0.06 a	8.92 ± 1.31 c	115.89 ± 44.35 b	35.97 ± 2.10 c	19.99 ± 2.16 c	271.77 ± 12.04 c	543.08 ± 26.45 cd
75 °C	1.57 ± 0.07 a	5.40 ± 0.51 ab	139.94 ± 89.08 bc	28.01 ± 2.66 b	20.61 ± 1.86 c	225.39 ± 24.22 bc	416.35 ± 22.58 b
100 °C	1.63 ± 0.04 a	4.65 ± 0.29 a	49.75 ± 0.83 a	16.53 ± 0.87 a	3.96 ± 0.36 a	200.94 ± 14.23 b	501.29 ± 5.23 c
125 °C	2.44 ± 0.98 ab	3.80 ± 0.26 a	37.24 ± 2.22 a	14.78 ± 0.69 a	4.00 ± 0.16 a	340.36 ± 4.55 de	624.92 ± 3.54 e
150 °C	1.36 ± 0.02 a	3.59 ± 0.13 a	36.82 ± 19.48 a	9.67 ± 1.71 a	2.74 ± 1.38 a	139.49 ± 10.59 a	291.03 ± 22.27 a
RT	cis-11-Eicosenoic acid (C20:1)	Linoleic acid (C18:2n6c)	Dihomo-γ linolenic acid (C20:3n6)	eicosatrienoic acid (C20:3n3)	Omega -6 (n-6)	Omega-3 (n-3)	(∑n-6)/(∑ n-3)
Control	27.73 ± 1.78 ab	52.70 ± 5.40 c	15.22 ± 14.09 c	0.62 ± 0.17 a	67.92 ± 15.59 cd	0.60 ± 0.18 a	113.77 ± 53.10 b
50 °C	35.99 ± 2.38 bc	52.46 ± 2.82 c	3.17 ± 0.92 a	1.81 ± 0.83 b	55.63 ± 2.90 c	1.81 ± 0.83 b	64.55 ± 27.93 ab
75 °C	30.68 ± 1.45 bc	43.06 ± 2.21 b	5.42 ± 1.02 b	3.63 ± 0.42 c	48.48 ± 2.80 bc	3.61 ± 0.42 c	13.74 ± 1.36 a
100 °C	30.50 ± 1.40 bc	30.41 ± 0.78 ab	0.55 ± 0.09 a	ND	30.96 ± 0.82 ab	ND	ND
125 °C	37.19 ± 1.7 c	22.02 ± 1.11 a	0.76 ± 0.09 a	ND	22.56 ± 1.29 a	ND	ND
150 °C	17.97 ± 1.90 a	29.63 ± 2.52 ab	0.09 ± 0.52 a	1.31 ± 0.45 ab	32.72 ± 2.33 ab	1.31 ± 0.45 ab	59.95 ± 37.5 ab
RT	∑ PUFA:∑ SFA	∑ SFA	∑ MUFA	∑ PUFA	∑ TFA		
Control	0.49 ± 0.04 ab	137.48 ± 22.48 c	959.68 ± 30.76 e	68.39 ± 15.58 dc	1165.55 ± 49.44 de		
50 °C	0.42 ± 0.08 ab	162.72 ± 47.72 d	861.84 ± 31.01 d	57.44 ± 3.16 c	1082.00 ± 68.90 d		
75 °C	0.49 ± 0.14 ab	174.91 ± 91.31 e	693.04 ± 8.73 c	52.11 ± 2.93 bc	920.06 ± 91.18 c		
100 °C	0.43 ± 0.01 ab	72.15 ± 1.16 b	518.69 ± 9.63 a	30.96 ± 0.82 a	621.80 ± 8.62 b		
125 °C	0.53 ± 0.03 b	58.26 ± 4.01 a	531.12 ± 6.06 ab	22.60 ± 1.05 a	419.06 ± 9.97 a		
150 °C	0.38 ± 0.12 a	82.89 ± 20.74 b	460.56 ± 16.97 b	34.03 ± 2.22 ab	577.48 ± 34.83 b		

RT (roasting temperature), ∑ (sum), SFA (saturated fatty acids), MUFA (monounsaturated fatty acids), PUFA (polyunsaturated fatty acids), PUFA:SFA (ratio of polyunsaturated fatty acids to saturated fatty acids), TFA (total fatty acids), ND (not detected). Values are the mean ± SE. Means within a column of the same parameter with different letters are significantly different (*p* < 0.001).

## Data Availability

Data is contained within the article.
